# Assessing the Protective Effects of Pioglitazone on Radiation-Induced Cardiac Injury in an *In Vivo* Model: A Biochemical and Histopathological Investigation

**DOI:** 10.2174/011573403X388076250807224407

**Published:** 2025-08-13

**Authors:** Fereshteh Talebpour Amiri, Fatemeh Jalali-Zefrei, Ehsan Zamani, Asma'a H. Mohamed, Aynaz Sourati, Mohammad Shourmij, Mehrsa Majdayeen, Arsalan Salari, Zobin Souri, Soghra Farzipour

**Affiliations:** 1 Department of Anatomy, Faculty of Medicine, Molecular and Cell Biology Research Center, Mazandaran University of Medical Sciences, Sari, Iran;; 2 Department of Radiology, Faculty of Medicine, Guilan University of Medical Science, Rasht, Iran;; 3 Department of Pharmacology and Toxicology, School of Pharmacy, Guilan University of Medical Sciences, Rasht, Iran;; 4 Department of Optometry Techniques, Technical College Al-Mussaib, Al-Furat Al-Awsat Technical University, Najaf, Iraq;; 5 Department of Radiotherapy and Oncology, Razi Hospital, Guilan University of Medical Sciences, Rasht, Iran;; 6 Razi Herbal Medicines Research Center, Lorestan University of Medical Sciences, Khorramabad, Iran;; 7 Cardiovascular Diseases Research Center, Department of Cardiology, Heshmat Hospital, School of Medicine, Guilan University of Medical Sciences, Rasht, Iran

**Keywords:** Irradiation, pioglitazone, heart injury, oxidative stress, fibrosis, radioprotective, ionizing radiation

## Abstract

**Introduction:**

RIHD is a significant complication in cancer radiotherapy, caused by oxidative stress and tissue damage. This study aimed to evaluate the protective effect of PGZ pretreatment against RIHD by assessing oxidative stress markers, enzyme levels, biochemical parameters, and cardiac tissue changes in a mouse model.

**Materials and Methods:**

72 BALB/c mice were randomly divided into eight groups: control, PGZ (10, 20, and 30 mg/kg), IR (8 Gy), and IR + PGZ (at three doses). PGZ was administered daily for 10 days before exposure to RT on Day 11. 24 hours post-irradiation, cardiac tissues were analyzed for MDA levels, GPX and GSH concentrations, and serum markers including LDH and CPK. Histopathological examination was performed at 1 week and 1 month after irradiation to evaluate early inflammatory changes and late fibrosis.

**Results:**

Results showed GPX activity increased by 28.2% and 48.4%, and GSH levels rose by 37.6% and 52.9% at doses of 20 and 30 mg/kg PGZ. MDA levels decreased by 40.35% and 52.63% at doses of 20 and 30 mg/kg, respectively. Serum LDH was reduced by 36.2% at 30 mg/kg. Tissue damage was significantly mitigated, with reductions of 88.9% at one week and 91.2% at one month. Fibrosis reduction was 23.5%, 41.5%, and 53.3% for 10, 20, and 30 mg doses.

**Discussion:**

The findings highlight PGZ's potential to protect against RIHD *via* antioxidant enhancement; however, further clinical validation and exploration of long-term safety are essential.

**Conclusion:**

PGZ shows promise in reducing RIHD by enhancing antioxidants and decreasing tissue damage, warranting further clinical investigation.

## 1 INTRODUCTION

IR induces injury in many tissues and organs. IR directly leads to abnormal cell function and metabolic disorders through its destructive effect on the molecular structure of cells. It also indirectly leads to DNA damage and further increases in cellular free radical levels through the production of free radicals and radiolytic decomposition of water. [1-3]. The damage by high-dose IR to biological macromolecules is fatal, and can lead to death within a short time, and the attack on DNA by the free radicals is the main cause of death at low and moderate radiation doses [4, 5]. RIHD is a growing clinical problem seen in cancer patients treated with thoracic radiotherapy and/or genotoxic chemotherapies. This disease comprises a wide spectrum of disorders, often with long latency periods between IR exposure and clinical presentation, requiring consistent follow-up and screening. Side effects of IR on the heart include pericarditis, CAD, arrhythmias, cardiomyopathy, valvular dysfunction, and heart failure. Pericarditis and pericardial effusions are potential short-term toxicities that may occur during or within the weeks following IR [6, 7]. There are several mechanisms involved in the induction of cardiotoxicity due to IR exposure in cancer patients, one of which is oxidative stress [8, 9]. In oxidative stress, an imbalance between the production of ROS and its removal by the antioxidant defense system mediates damage to the structure of cellular macromolecules, including lipids, proteins, and DNA [10, 11].

ROS generated during radiation exposure play a central role in the development of tissue fibrosis. Excess ROS cause oxidative damage to cellular structures, including DNA, lipids, and proteins, leading to activation of fibrogenic signaling pathways such as TGF-β. This promotes the transdifferentiation of fibroblasts into myofibroblasts, which produce excessive extracellular matrix components, resulting in tissue stiffening and fibrosis. Therefore, oxidative stress driven by ROS is a key mechanistic mediator in radiation-induced tissue remodeling and fibrosis [12, 13].

Oxidative stress and chronic and acute overproduction of ROS have always been considered to be important pathophysiological mediators leading to CVD [14, 15]. Radiosensitizers and radioprotectors have shown the potential to mitigate cardiotoxicity and tissue damage during TBI. These agents can either boost radiation's effectiveness on tumors or safeguard healthy tissues from radiation-related damage, leading to better patient outcomes [16, 17]. However, few of these compounds have clear efficacy and superior performance. Researchers have shown that PGZ administration can significantly reduce radiation-induced ROS production and increase antioxidant enzyme activity, thereby regulating cell-protective proteins in cardiac tissue [5, 18]. In addition, PGZ modulates proinflammatory cytokines and signaling cascades with anti-inflammatory properties, which may help reduce the inflammatory response associated with radiation injury to the heart [19]. Moreover, numerous studies have demonstrated the cardioprotective effects of PGZ in various experimental models. It exerts its protective effects primarily through activation of the PPARγ, which regulates gene expression related to metabolism, inflammation, and fibrosis. Activation of PPARγ by PGZ leads to downstream signaling involving the MAPK pathway, particularly ERK1/2, which promotes cell survival and reduces apoptosis. Additionally, PGZ modulates inflammatory mediators by decreasing cyclooxygenase-2 (COX-2) expression. Its ability to reduce oxidative stress and inflammatory responses helps limit tissue damage and fibrosis in the myocardium, thereby improving overall cardiac function [20, 21]. These data suggest that the antioxidant and anti-inflammatory effects of PGZ can be used to protect against heart injury induced by ionizing radiation, which is potentially beneficial for patients undergoing IR or those injured in nuclear accidents. The detrimental effects of IR on the heart can be acutely apparent in the first week or delayed after several weeks or months. Acute radiation-induced cardiac damage is characterized by oxidative stress, inflammation, and structural changes, while delayed effects can lead to long-term complications such as fibrosis and impaired cardiac function. Evaluating the radioprotective potential of pharmacological interventions at the early and delayed stages is crucial to fully understanding their efficacy in mitigating the multifaceted cardiac complications associated with radiation exposure. According to the above, PGZ may contribute to its potential radioprotective effects in the heart by effectively suppressing oxidative stress and inflammation, two key pathways underlying the detrimental effects of ionizing radiation exposure. In this study, we investigated the ability of PGZ, with potent antioxidant and anti-inflammatory properties, to protect the heart against RIHD one week and one month after X-ray irradiation in a murine model. By assessing the effects of PGZ on oxidative stress markers, inflammatory responses, and histopathological changes in the cardiac tissue, we aim to elucidate the radioprotective mechanisms of this pharmacological agent, which may have important implications for patients undergoing radiotherapy or populations affected by nuclear accidents.

## 2 MATERIALS AND METHODS

### 2.1 Chemicals

The PGZ, along with all the necessary reagents for buffer preparation were purchased from Sigma-Aldrich and Merck Companies. Masson's trichrome, hematoxylin, and eosin stains were purchased from Merck (Germany).

### 2.2 Animals

In this study, 72 male BALB/c mice (body weight: 25-30 g; age: 6-8 weeks) were obtained from the animal research facility at the School of Pharmacy. Before the experiment, the mice were carefully acclimatized in the same facility for one week under uniform conditions to reduce stress and ensure adaptation. These conditions included a 12-hour light/dark cycle, a temperature of 24 ± 2°C, and a relative humidity of 52%. During the study, the mice were provided with a standard diet and adequate water and maintained under conventional laboratory conditions to promote full acclimation and minimize stress.

### 2.3 Ethics Declarations

The ethical approval code IR.GUMS.AEC.1401.005 for this experimental research was from the Guilan University of Medical Sciences. Furthermore, this study follows the ARRIVE guidelines 2.0. The experiments were carried out in accordance with the established principles outlined in the “Guide for the Care and Use of Laboratory Animals” prepared by the National Academy of Sciences and published by the National Institutes of Health [22]. In this study, mice were euthanized using carbon dioxide, and to ensure death was achieved, a reflex test was performed to confirm that no pain or distress was inflicted on the animals.

### 2.4 Study Design

The number of mice in each group was determined based on previous studies. During the experiment, especially after radiation exposure, any animals that showed issues such as loss of appetite, weight loss, or abnormal movement were excluded from the study following the advice of a veterinarian and a laboratory animal specialist. The mice were then randomly divided into 8 groups (9 animals/group) [23]:

1. Control group: Mice received distilled water + CMC 0.5% (as the vehicle for PGZ) for 10 consecutive days.
2. PGZ group (10 mg/kg): Mice received PGZ at a 10 mg/kg dose.
3. PGZ group (20 mg/kg): Mice received PGZ at a 20 mg/kg dose.
4. PGZ group (30 mg/kg): Mice received PGZ at a 30 mg/kg dose.
5. IR group (8 Gy): Mice were exposed to 8 Gy total body irradiation on the 11^th^ day.
6. IR + PGZ (10 mg/kg) group: Mice received PGZ at a 10 mg/kg dose and were exposed to 8 Gy.
7. IR + PGZ (20 mg/kg) group: Mice received PGZ at a 20 mg/kg dose and were exposed to 8 Gy.
8. IR + PGZ (30 mg/kg) group: Mice received PGZ at a 30 mg/kg dose and were exposed to 8 Gy.

PGZ was dissolved in distilled water with 0.5% carboxymethyl cellulose to ensure a uniform suspension. Doses of 10, 20, and 30 mg/kg were administered to groups of 18 mice each, with doses calculated based on individual weights. The drug solution was prepared at a volume of 0.2 ml per mouse, multiplied by 1.5 to ensure sufficient volume and prevent errors during administration. This procedure was carried out continuously for 10 days [24, 25].

### 2.5 Irradiation Procedure

A Plexiglas enclosure was specially designed, featuring 18 separate compartments to house the mice individually for the animal irradiation procedure. On day 11 of the study, the mice were exposed to a total body X-ray dose of 8 Gy, using a 6 MeV X-ray beam generated by a clinical linear accelerator (Siemens Primus, Germany) at a dose rate of 2 Gy per minute. The mice were not anesthetized during this IR procedure. Immediately after the IR exposure, the mice were released from the cages and returned to their previous standard housing conditions [26, 27].

### 2.6 Sample Collection

24 hours after the IR, 3 to 5 mice from each group were euthanized using carbon dioxide to conduct biochemical tests. After confirming their death through reflex tests and cessation of heartbeat, the hearts were harvested, homogenized in a mannitol buffer, and centrifuged. The supernatant fluid was collected and used for biochemical analyses. The samples were stored at -80°C until the time of testing. To evaluate the early and delayed effects of IR on the heart tissue in the irradiated mice, histopathological assessments were conducted at two separate time points. The first assessment was carried out one week after the radiation exposure. The second assessment was performed 30 days after the IR, using tissue samples from the mice [28].

### 2.7 Biochemical Assay

#### 2.7.1 MDA Analysis

The protein concentration was determined at the start of each experiment. The protein level of the heart homogenate supernatant was measured using the Bradford method. This colorimetric assay is based on an absorbance shift of the Coomassie Brilliant Blue G-250 dye, which is detected at 595 nm. The evaluation of MDA was performed using the Nalondi Kit (Navand Salamat Company, Iran). The thiobarbituric acid (TBA) in this kit was employed to assess lipid peroxidation. The procedure was as follows: A solution containing the sample or standard (200 μl) was prepared with the working solution (800 μl). The sample was incubated at 95°C for 45 minutes. After the sample was cooled for 15 minutes, centrifugation was performed at 3000 rpm for 10 minutes. The reaction between MDA and TBA produced a red fluorescent compound at a wavelength of 550 nm. MDA content was expressed as µM [29].

#### 2.7.2 GSH Analysis

The assessment of GSH levels was performed using the Nargol kit (Navand Salamat Company, Iran). The procedure was as follows: The sample was mixed with DTNB and glutathione reductase and incubated for 30 seconds. Then, a cofactor solution was added. The conversion of reduced glutathione to glutathione disulfide effectively converts hydrogen peroxide to water, and subsequently, glutathione reductase regenerates reduced glutathione from glutathione disulfide. This process was carried out, and the absorbance was measured at 412 nm to quantify the GSH levels. GSH content was expressed as µM [30].

#### 2.7.3 GPX Analysis

The assay is based on the oxidation of NADPH, which is monitored spectrophotometrically at 340 nm. First, a 1 mM NADPH standard solution is prepared, and serial dilutions are made to create a calibration curve. The assay procedure involves adding 50 μL of the heart supernatant sample or standard dilutions to a 96-well plate, followed by 40 μL of preprepared Reagent 1. After a 15-minute incubation at room temperature, 10 μL of reagent 2 is added to initiate the reaction. The optical density is measured immediately at 340 nm (time 0) and again after 5-10 minutes of incubation. The standard curve is plotted using the time 0 optical density values of the standard dilutions. The GPX activity in the heart tissue sample is then calculated and expressed in mU/mL based on this standard curve. The provided method for the determination of glutathione peroxidase (GPX) activity is based on the protocol from the NAGPIX GPx Assay Kit (Navand Salamat Company, Iran) [31].

### 2.8 Serum LDH and CPK Measurement

The activity of lactate dehydrogenase (LDH) in the serum samples was determined using an enzymatic colorimetric kit (Pars Azmoon Co., Tehran, Iran). This method measures the rate of oxidation of NADH by LDH. The absorbance change per minute was detected at 340 nm using a spectrophotometric instrument (Analytik Jena Specord 50 Plus), and the results were expressed as U/L. The activity of serum creatine phosphokinase (CPK) was measured using an enzymatic colorimetric kit (Pars Azmoon Co., Tehran, Iran). This method relies on the principle that creatine kinase catalyzes the conversion of creatine into ADP and phosphocreatine. The absorbance change per minute was detected at 340 nm, and the results were expressed as U/L [32].

### 2.9 Histopathological Assessments

Three to four heart tissue samples were fixed in 10% formalin, then dehydrated in a series of alcohols, clarified in xylene, and embedded in paraffin for routine histological sectioning. Sections measuring 5 μm in thickness were stained with hematoxylin and eosin (H&E) and Masson's trichrome staining according to standard protocols. Subsequently, the histopathological slides were evaluated under light microscopy (Olympus, Japan) with a magnification of 20×. Sections were analyzed blindly by a histologist. The extent and severity of inflammation, interstitial proliferation, myocyte necrosis, myocyte vacuolization, edema, congestion, and hemorrhage in a field were used to score the heart injury on a scale from 0 to 4. A score of 0 was assigned to a normal heart, 1 for minimal lesions, 2 for mild, 3 for moderate, and 4 for severe injury. For the semi-quantitative evaluation, eight slides from each sample were examined under a light microscope, with five fields evaluated per slide. The mean scores were then calculated for each heart sample in the groups. Masson's trichrome staining was used to evaluate the extent of myocardial fibrosis in the hearts of irradiated mice compared to non-irradiated controls. This staining technique effectively highlighted the increased collagen deposition in the myocardial interstitium of irradiated hearts, indicating the development of significant myocardial fibrosis as a result of irradiation-induced cardiac injury over time. The increased collagen staining with Masson's trichrome in irradiated hearts compared to non-irradiated controls allowed for the quantitative assessment of radiation-induced myocardial fibrosis [23].

### 2.10 Statistical Analysis

The mean ± SEM was obtained after data analysis. One-way ANOVA and Tukey’s post hoc test were utilized to determine the differences between the groups. Statistical significance was indicated by *p*<0.05. Prism 8 software was used for analysis and graph preparation.

## 3 RESULTS

### 3.1 Effect of PGZ on Oxidative Stress and Antioxidant Markers of Cardiac Tissue

**Fig. (1):**
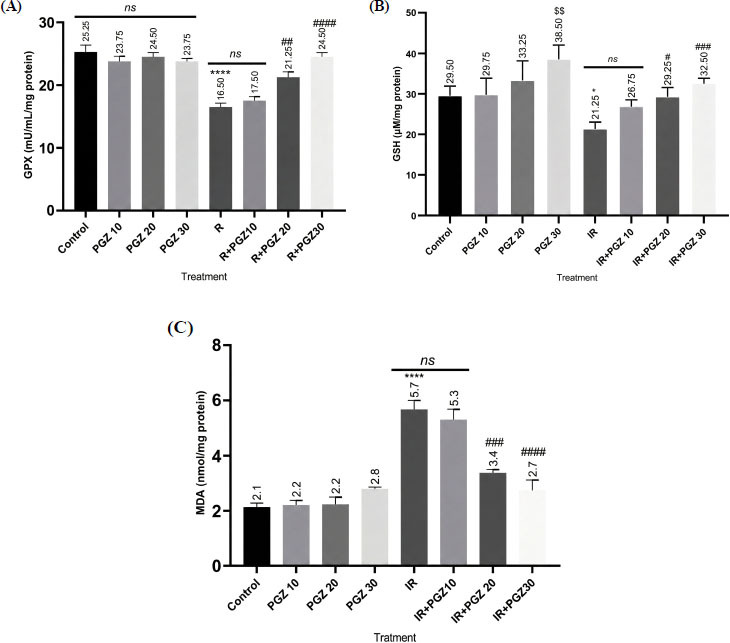
Antioxidant and oxidative parameters in heart tissue of irradiated mice. (**A**) GPX levels of heart tissue; ****p<0.0001 *vs* control, ^##^*p*<0.01 *vs* R, ^####^*p*<0.0001. (**B**) GSH levels of heart tissue; *p<0.05 *vs* control, ^#^*p*<0.05 and ^###^*p*<0.001 *vs* R. (**C**) MDA levels of heart tissue; ****p<0.0001 *vs* control, ^###^*p*<0.001 and ^####^*p*<0.0001 *vs* R. Data are displayed as Mean ± SEM.

This study investigated the effects of PGZ on oxidative stress and antioxidant markers in the cardiac tissue of irradiated mice. Considering the key endogenous antioxidant effect of GPX and GSH *versus* oxidative agents, the levels of these markers were investigated in the homogenized cardiac tissue samples. As demonstrated in Fig. (**1A**), IR significantly decreased GPX levels in irradiated mice compared with the control group (*p<*0.0001,16.5± 0.64 *vs* 25.25 ± 1.10) but pretreatment with PGZ in two doses of 20 mg/kg and 30 mg/kg significantly increased GPX levels compared with irradiated mice (*p<*0.01 and *p<*0.0001,21.25±0.85 and 24.5±0.64 *vs* 16.5 ± 0.64, respectively). This experiment demonstrates that pretreatment with PGZ in doses of 20 and 30 mg/kg significantly reversed the decrease in GPX levels in the cardiac tissue of irradiated mice. The 10 mg/kg dose decreased, but this decrease was not significant. GSH, as another endogenous antioxidant factor was significantly reduced in irradiated mice compared to the control group (*p<*0.05 *vs* control, 21.25 ± 0.85 *vs* 29.5 ± 1.5), but pretreatment with PGZ in doses of 20 and 30 mg/kg significantly (*p<* 0.05 and *p*<0.0001,21.25±1.78 *vs* 29.25±1.1 and 32.5±0.64) increases its levels in cardiac tissue of irradiated mice (Fig. **1B**). Furthermore, IR significantly increased the level of MDA compared to the control group (5.7± 0.32 *vs* 2.1± 0.14). Pre-treatment with PGZ at 20 and 30 mg/kg doses reduced this oxidative stress factor (3.4± 0.12 and 2.7±0.38 *vs* 5.7 ±0.32). (Fig. **1C**).

### 3.2 Assessment of PGZ on Blood Parameters: Creatine Phosphokinase and Lactate Dehydrogenase

**Fig. (2):**
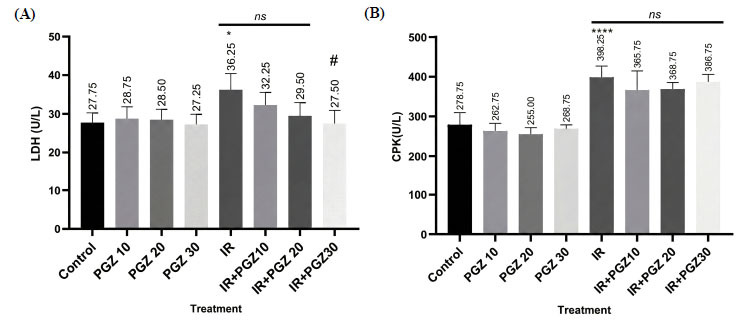
(**A**) Lactate dehydrogenase (LDH) in irradiated mice and (**B**) Serum creatinine phosphokinase (CPK). Values are described as Means ± SEM. ****p <0.0001 *vs* control, *p <0. 05 *vs* control and ^#^*p*<0.05 *vs* R.

The analysis of creatine phosphokinase (cpk) and lactate dehydrogenase (LDH) levels in the serum of irradiated mice showed that radiation significantly increased (*p*<0.05, 36.25 ±2.09 *vs* 27.75± 1.25) LDH levels in the serum of irradiated mice compared to the control group, but pretreatment with PGZ at a dose of 30 mg/kg significantly decreased LDH levels (*p*<0.05, 28.75 ± 1.25 *vs* 36.25 ± 2.09). In contrast, lower doses of PGZ (10 and 20 mg/kg) did not produce significant changes in the serum LDH content and showed no difference compared to the irradiated group. These lower PGZ doses were unable to effectively reduce the elevated LDH levels caused by radiation exposure (Fig. **2A**). In addition to the effects on LDH, the analysis also revealed that IR exposure significantly increased the serum CPK levels (*p*<0.0001, 398.3±14.37 *vs* 278±15.33) in the irradiated mice compared to the control. However, in contrast to the effects on LDH, pretreatment with PGZ at any of the tested doses did not significantly decrease the elevated CPK levels caused by radiation exposure (Fig. **2B**).

### 3.3 Histological Evaluation of Cardiac Tissue One Week and 30 Days After Irradiation

**Fig. (3):**
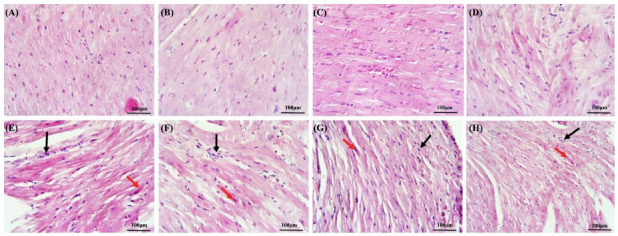
Photomicrographs show the effect of PGZ and 8 Gy IR exposure on the histological architecture of the heart at one week post-irradiation. (**A**) Control; heart tissue in normal histological structure, (**B-D**): PGZ treated with different doses (10, 20 and 30 mg/kg); heart structure similar to the control group, (**E**) IR; Heart structure shows infiltration of inflammatory cells (black arrow), myocyte eosinophilic (Red arrow), (**F-H**) PGZ+IR; Administration of PGZ improved this injury. H&E staining; 20× magnification; Scale bar = 100 µm. IR; irradiation; PGZ; pioglitazone.

**Fig. (4):**
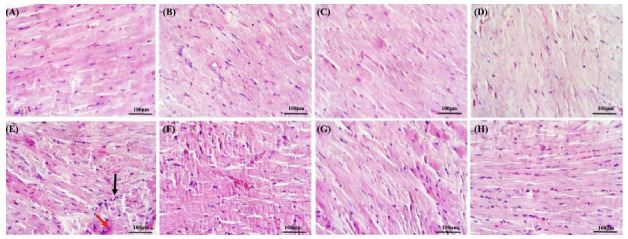
Photomicrographs show the effect of PGZ and 8 Gy IR exposure on the histological architecture of the heart at one month post-irradiation. (**A**) Control; heart tissue in normal histological structure, (**B-D**): PGZ treated with different doses (10, 20 and 30 mg/kg); heart structure similar to the control group, (**E**) IR; Heart structure with infiltration of inflammatory cells (black arrow) and myocyte eosinophilic that these changes were reduced compared to one week after IR. (Red arrow), (**F-H**) PGZ+IR; Administration of PGZ improved this injury. H&E staining; 20× magnification; Scale bar = 100 µm. IR; irradiation; PGZ; pioglitazone.

Histomicrostructural evaluation of the heart showed normal structure in the control group. The PGZ-alone groups showed a cardiac tissue structure similar to the control group, without any significant pathological changes (Figs. **3A**-**D**). This indicates that PGZ alone did not exhibit detrimental effects on the microscopic anatomy of the heart. In contrast, the IR group displayed prominent histopathological alterations. These included intense infiltration of inflammatory cells, appearing as dark purple granules in the cardiac tissue. Additionally, hemorrhage, edema, cytoplasmic vacuolation, myocyte degeneration, and necrosis were evident in this group (Fig. **3E**). These findings demonstrate that IR induced substantial cardiac tissue damage. Interestingly, the group that received PGZ pretreatment in doses of 10, 20, and 30 mg/kg showed a marked reduction and amelioration of the irradiation-induced pathological changes (Figs. **3F**-**H**). Moreover, increasing the dose of PGZ further enhanced the attenuation of these histopathological manifestations. These histopathological alterations observed one week after IR indicate an acute inflammatory and degenerative response of the cardiac tissue to the radiation. The presence of these significant pathological sites suggests that IR induced substantial and rapid damage to the myocardial structure within the first week of exposure. Interestingly, the follow-up evaluation conducted one month after IR demonstrated preserved structural integrity in the control group and in all three doses of PGZ, similar to the tissues one week after IR (Figs. **4A**-**D**). However, the extent of myocyte damage and inflammatory infiltration in the irradiated mice had decreased compared to the findings at one week (Fig. **4E**). This suggests that the cardiac tissue exhibited some recovery and healing processes over the longer time frame following the initial radiation insult. Importantly, the pretreatment with PGZ, at all three tested doses, effectively mitigated the irradiation-induced histopathological changes at the one-week and one-month time points (Figs. **4F**-**H**). The higher doses of PGZ showed enhanced attenuation of these pathological manifestations compared to the lower doses (Fig. **4H**).

The semi-quantitative evaluation of the scoring system is shown in Fig. (**5**). Fig. (**5A**) shows that radiation on the 7th day caused significant damage to the heart tissue compared to the control group (2.9±0.74 *vs* 0.33±0.52; *p*<0.0001). PGZ administration at two doses of 20 and 30 mg/kg was able to significantly reduce the severity of damage (*p*<0.001). The semi-quantitative evaluation of the scoring system in Fig. (**5B**) showed that the severity of heart tissue damage after 30 days was slightly reduced compared to the first week. However, this difference was still significant compared to the control group (2.5±0.53 *vs* 0.22±0.44; *p*<0.0001). Also, receiving PGZ at two doses of 20 and 30 mg/kg was able to significantly reduce the severity of damage. However, the effect of the 30 mg/kg dose was more effective than the 20 mg/kg dose, as seen on the seventh day.

### 3.4 Masson's Trichrome Staining Evaluation 30 Days After Irradiation

**Fig. (6):**
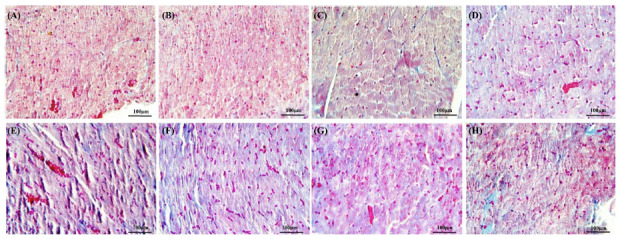
Photomicrographs show the effect of PGZ and 8 Gy IR exposure on collagen deposition of the heart at one-month post-irradiation. (**A**) Control, (**B-D**): PGZ treated with different doses (10, 20, and 30 mg/kg), (**E**) IR; Blue dots indicate fibrosis of the heart structure, (**F-H**) PGZ+IR; Administration of PGZ reduced the severity of fibrosis. Masson’s trichrome staining; 20× magnification; Scale bar = 100 µm. IR; irradiation; PTZ; Pioglitazone.

Masson’s trichrome staining analysis, performed one month after irradiation exposure, provided further insights into the cardiac tissue changes (Fig. **6**). The control group (Fig. **6A**) showed a normally distributed collagen. Similarly, Figs. (**6B**-**D**) also displayed a normal distribution of collagen, consistent with the control group. In the group that received IR alone (Fig. **6E**), Masson’s trichrome staining revealed a significant increase in collagen deposition within the myocardium. This finding indicates the presence of severe fibrosis in the cardiac tissue of the irradiated mice at the one-month time point. In contrast, pretreatment with PGZ (Figs. **6F**-**H**), particularly at the higher doses) effectively prevented the irradiation-induced fibrotic changes in the myocardium. The cardiac tissue in the PGZ pretreatment groups exhibited reduced collagen accumulation compared to the IR alone group. These results, obtained through targeted Masson's trichrome staining, demonstrate that the IR induced profound fibrotic remodeling of the cardiac tissue over the longer term. However, pretreatment with PGZ was able to mitigate the development of this radiation-induced cardiac fibrosis in a dose-dependent manner. Fig. (**7**) illustrates the extent of heart fibrosis observed on the 30^th^ day across different experimental groups. The results indicate that the group subjected to IR exhibited the highest level of fibrosis compared to the control group, with statistical significance levels.

## 4 DISCUSSION

RIHD from TBI has been extensively studied, revealing significant early and late effects on cardiac function. Research indicates that IR leads to persistent DNA damage, fibrosis, and functional impairments in cardiac tissue, which can be detected through various methodologies. The present study utilized a multifaceted approach to investigate the protective effects of PGZ pretreatment against irradiation-induced cardiac damage in a mouse model.

The biochemical analyses revealed that IR exposure significantly decreased the levels of the endogenous antioxidant enzymes GPX and GSH in the cardiac tissue. Pretreatment with PGZ, at the 20 mg/kg and 30 mg/kg doses, was able to effectively reverse the irradiation-induced depletion of these key antioxidant markers. The biochemical analyses also revealed that IR exposure significantly increased lipid peroxidation in cardiac tissue. Pretreatment with PGZ at doses of 20 mg/kg and 30 mg/kg was able to effectively reverse the radiation-induced increase in lipid peroxidation.

Our results were concordant with the findings of the study, which reported a significant decrease in GSH and an increase in MDA following IR in the cardiac tissue of Albino Wistar Rats [33]. Studies indicate that TBI results in a chronic decrease in antioxidant levels, including GSH and GPX, which persists long after exposure. This depletion contributes to increased oxidative stress, which can result in DNA damage and cellular dysfunction in normal cells. Researchers found that chronic GSH depletion significantly exacerbated ventricular remodeling and dysfunction in a mouse model of pressure overload, and this effect was reversed by GSH supplementation.

Our study showed a significant increase in GSH and GPx levels following IR in the PGZ pretreatment group, consistent with the established role of these antioxidants in mitigating irradiation-induced oxidative stress. This increase in GSH is in line with previous studies demonstrating PGZ's ability to elevate GSH levels in various models of oxidative stress. Studies in diabetic rabbits [34] and high-fat diet models [35] have shown similar increases in GSH levels following PGZ treatment, supporting its role in enhancing antioxidant defenses. Furthermore, our study demonstrated a significant reduction in radiation-induced MDA levels in the PGZ pretreated groups (both 20 mg and 30 mg). This finding strongly suggests that PGZ's protective effects extend to mitigating lipid peroxidation, a major contributor to radiation-induced damage. This observation corroborates previous findings showing PGZ's ability to significantly lower MDA in various oxidative stress models, such as metabolic syndrome models and isoprenaline-induced myocardial infarction models [36, 37]. Because elevated LDH and CPK, as markers of cellular damage and metabolic alterations after chemotherapy and IR, are associated with cardiotoxicity [38], in this study, both of these markers were evaluated in the serum content of different groups of mice. Our findings showed a rapid increase in both CPK and LDH within 24 hours after IR. This contrasts with previous studies reporting minimal CPK elevation at moderate IR, suggesting dose-dependent effects. However, our results are consistent with an animal model where TBI resulted in a sudden increase in serum CPK activity in X-irradiated rats [39]. Pretreatment with 30 mg/kg of PGZ significantly decreased serum LDH levels in irradiated mice, while there was no reduction in CPK levels observed at any dose of PGZ. LDH is a general marker of cellular damage and metabolic disturbance, while CPK—particularly its MB isoenzyme—is more specific to cardiac muscle injury. Therefore, the reduction in LDH suggests that PGZ effectively alleviates overall cytolytic and metabolic stress induced by irradiation at this dose. However, the unchanged CPK levels may indicate that specific cardiac injury either is less responsive or requires longer treatment duration or different dosing to observe a protective effect. These findings were consistent with previous studies showing that PGZ significantly decreased LDH levels in diabetic animals at doses of 1 and 10 mg/kg, while at 100 mg/kg, it significantly increased CPK levels, indicating potential cardiac risk [40]. These findings suggest that while PGZ can reduce cytolytic damage in T2DM, its potential to increase cardiac damage at higher doses warrants careful monitoring. This is particularly important for patients with existing heart conditions.

At one week post-irradiation, significant cardiac tissue injury, as evidenced by inflammatory cell infiltration, hemorrhage, edema, myocyte degeneration, and necrosis, was observed in the IR alone group. These observations were consistent with the study by Shigemori *et al.,* which found persistent DNA damage response and macrophage repolarization, diffuse inflammation, and vacuolization at one week post-IR [41]. At the one-month time point, the extent of myocyte damage and inflammatory infiltration had decreased, suggesting some degree of cardiac tissue recovery. This finding was in line with Lee *et al.'s* study, who observed that no significant change was observed in the pathological analysis of the cardiac tissue one month after radiotherapy with 0 to 10 Gy [42]. However, they did not examine the early effects of radiation one week after IR. Furthermore, trichrome staining also revealed a significant increase in collagen deposition within the myocardium. This finding indicates the presence of severe fibrosis in the cardiac tissue of the IR group at the one-month time point. This observation aligns with existing literature that highlights radiation-induced myocardial fibrosis (RIMF) as a critical concern following IR. While previous studies have documented significant increases in collagen volume fraction as early as 14 days post-irradiation, the specific timeline for collagen deposition at one month remains underexplored in the literature. For instance, one study reported a notable increase in collagen volume fraction from 3.76% to 22.05% within the first two weeks post-irradiation (*p=*0.003), emphasizing the rapid onset of fibrotic changes. However, the absence of detailed data regarding the one-month mark suggests a gap in understanding the progression of RIMF. This is particularly important as RIMF is recognized as a major risk factor for long-term cardiac complications, including heart failure and arrhythmias [43, 44].

The mechanisms underlying this collagen deposition are multifaceted, involving an inflammatory response characterized by the infiltration of immune cells and the release of pro-inflammatory cytokines, which promote fibrosis. Additionally, the activation of fibroblasts post-radiation leads to excessive collagen synthesis, contributing to myocardial stiffness and functional impairment [13, 45].

One month after IR, while there was a noticeable reduction in inflammation and necrosis—evident from hematoxylin and eosin staining—significant collagen deposition and fibrosis were still present in the cardiac tissue. This finding highlights the complex nature of the healing process following IR, where the inflammatory response may subside, but fibrotic changes continue to develop [46, 47]. These results underscore that, despite improvements in inflammation at the one-month mark, the presence of fibrosis poses potential long-term risks for cardiac health. This aligns with existing literature, emphasizing the importance of monitoring for RIMF as a critical concern in RIHD [48]. Despite this structural damage to the heart tissue, pretreatment with PGZ in.

The three doses of 10, 20, and 30 mg/kg showed a marked reduction and amelioration of the radiation-induced pathological changes in irradiated groups. Moreover, increasing the dose of PGZ further enhanced the attenuation of these histopathological manifestations. These findings were in accordance with many studies that suggested the protective effect of PGZ against inflammation and damage in cardiac tissue following oxidative stress [36, 49, 50]. Fibrosis after IR results from fibroblast activation, excess collagen deposition, and extracellular matrix accumulation, leading to tissue stiffening and impaired cardiac function [13]. PGZ mitigates fibrosis by activating PPAR-γ receptors, reducing inflammation, and fibroblast proliferation. It also downregulates pro-fibrotic cytokines like TGF-β, thereby inhibiting collagen synthesis and fibrotic progression [51, 52].

PGZ offers radioprotective effects against RIHD through multiple mechanisms. It enhances antioxidant defenses by increasing levels of GSH and GPX4, reducing oxidative stress, lipid peroxidation, and inhibiting ferroptosis—a regulated form of cell death linked to lipid peroxidation [53]. Moreover, its anti-inflammatory properties help mitigate radiation-induced inflammation, preserving cardiac tissue integrity. Besides ferroptosis, other cell death pathways, such as apoptosis and necrosis, also contribute to cardiac damage. PGZ’s antioxidant and anti-inflammatory effects help protect against these mechanisms as well.

Various studies have shown that the use of antioxidant and anti-inflammatory compounds can help reduce RIHD. PGZ, as a PPAR-γ agonist, exerts significant protective effects on cardiac tissues by decreasing oxidative stress, inflammation, and fibrosis. This broad range of effects makes PGZ highly effective against radiation-induced damage. On the other hand, N-acetylcysteine (NAC), a precursor to GSH and a direct antioxidant, plays an important role in cardiomyocyte protection by scavenging free radicals and maintaining mitochondrial function, although it has limited capacity to repair radiation-induced DNA damage [54, 55]. Similarly, gliclazide, known for its antioxidant and anti-inflammatory properties, has been shown in animal studies to reduce oxidative damage and histopathological changes in the heart and lungs following IR, thereby preserving tissue structure [23]. Hesperidin, a natural flavonoid, enhances antioxidant enzyme activity and reduces oxidative markers, inflammation, and fibrosis in cardiac tissue, although its effects on certain structural damages, such as thrombosis and myocardium degeneration, are limited [56]. Considering the complementary mechanisms of these compounds, it can be concluded that PGZ, due to its multifaceted effects, especially in reducing inflammation and fibrosis, is a key agent for cardiac radioprotection, while NAC, gliclazide, and hesperidin mainly contribute through their potent antioxidant capacities. The combination or strategic selection of these agents may provide an effective approach to mitigate radiation-induced heart injury.

Future investigations should prioritize establishing the optimal dosing, timing, and safety profile of PGZ for cardioprotection in patients undergoing radiotherapy. Randomized controlled trials are essential to validate its efficacy across various cancer types, radiation protocols, and patient populations, while carefully assessing potential cardiovascular risks at higher doses. Clinically, prophylactic administration of PGZ at doses around 20–30 mg/kg before radiation exposure could offer a promising strategy for high-risk patients, particularly those with comorbid cardiovascular disease. Regular monitoring of key biomarkers—such as GSH, GPX, lipid peroxidation, and inflammatory markers—alongside functional imaging, would be critical to tailor therapy and prevent long-term cardiac complications. Caution is warranted with dose escalation, as excessive PGZ may pose risks of adverse cardiac effects, especially in individuals with existing heart disease. Future research should also explore combination approaches, integrating PGZ with other antioxidants like NAC or natural agents such as hesperidin, aiming for synergistic cardioprotection. Ultimately, translating these promising preclinical results into routine clinical practice will depend on rigorous clinical trials, standardized administration protocols, and personalized risk stratification, paving the way for safer, more effective management of radiation-induced heart disease in cancer patients. Due to time and financial constraints, we were unable to investigate the genes involved in the inhibition of ferroptosis that could have provided protective effects against radiation. On the other hand, the follow-up period in this study was limited to one month, and evaluating the long-term effects of PGZ would be valuable.

## CONCLUSION

This study demonstrates that PGZ has significant protective effects against RIHD resulting from TBI. Our findings show that pretreatment with PGZ effectively mitigates oxidative stress and lipid peroxidation associated with IR. This is evidenced by the restoration of antioxidant enzyme levels (GPX and GSH) and a reduction in MDA levels in cardiac tissue. These biochemical improvements correlate with a decrease in markers of cellular damage, such as LDH, highlighting PGZ’s role in preserving cardiac health. Histopathological analyses further indicate that PGZ pretreatment alleviates RIHD, including inflammatory cell infiltration and fibrosis. This suggests that PGZ not only promotes recovery but also helps to prevent the progression of RIHD. This aligns well with existing literature regarding the protective effects of PGZ against oxidative stress and inflammation, underscoring its potential as a therapeutic agent for patients undergoing IR. While our results are promising, there are some limitations to the study, including the short follow-up period and the lack of tissue analysis related to the inhibition of ferroptosis. These limitations suggest that further research is necessary to fully unveil the mechanisms underlying PGZ’s cardioprotective effects. Future studies should explore long-term outcomes and the molecular pathways involved in mediating these protective effects, especially concerning ferroptosis. Overall, this research contributes to the understanding of potential interventions for alleviating cardiac damage in patients receiving IR, emphasizing the need for careful monitoring and management of RIHD.

## AUTHORS’ CONTRIBUTIONS

The authors confirm their contribution to the paper as follows: Study conception and design: FJZ and SF. Methodology: FTA, EZ, AS and MM. Validation: AS. Draft manuscript: AHM, MS and ZS. All authors reviewed the results and approved the final version of the manuscript.

## Figures and Tables

**Fig. (5):**
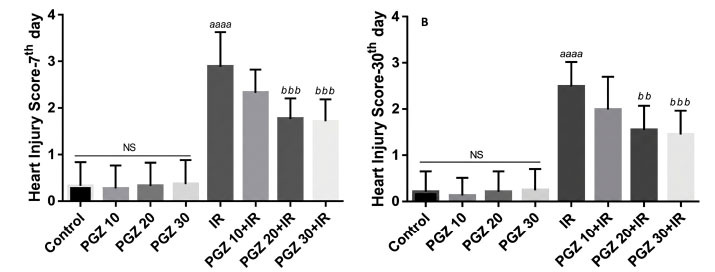
The heart injury score on the 7^th^ and 30^th^ days. Values are described as Means ±SD. The highest score was for the IR group. (**A**) Significant *vs* control, (**B**) Significant *versus* IR group. IR: irradiation, PGZ: pioglitazone.

**Fig. (7):**
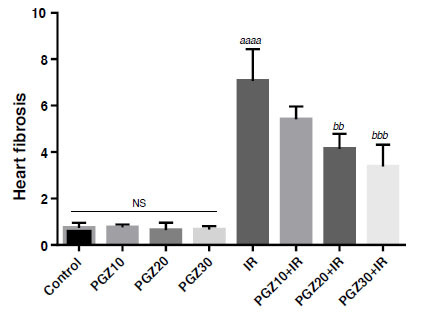
The heart fibrosis on the 30^th^ day. Values are described as Means ±SD. The most fibrosis was for the IR group. *^aaaa^p<*0.0001 *vs* control, *^bb^p*<0.01 *vs* IR, *^bbb^p<*0.001 *vs* IR. NS: non-significant, IR: Irradiation, PGZ: pioglitazone.

## Data Availability

The data that support the findings of this study are available from the corresponding author upon reasonable request.

## LIST OF ABBREVIATIONS

CAD: Coronary Artery Disease
COX-2: Cyclooxygenase-2
CPK: Creatinine Phosphokinase
CVD: Cardiovascular Disease
GPX: Glutathione Peroxidase
GSH: Glutathione
LDH: Lactate Dehydrogenase
MDA: Malondialdehyde
NADPH: Nicotinamide Adenine Dinucleotide Phosphate Hydrogen
PGZ: Pioglitazone
PPARƔ: Proliferator-activated Receptor Gamma
RIHD: Radiation-Induced Heart Damage
ROS: Reactive Oxygen Species
TBI: Total Body Irradiation
